# Heme Oxygenase 1 and 2 Differentially Regulate Glucose Metabolism and Adipose Tissue Mitochondrial Respiration: Implications for Metabolic Dysregulation

**DOI:** 10.3390/ijms21197123

**Published:** 2020-09-27

**Authors:** Hongwei Yao, Abigail L. Peterson, Jie Li, Haiyan Xu, Phyllis A. Dennery

**Affiliations:** 1 Department of Molecular Biology, Cell Biology & Biochemistry, Division of Biology and Medicine, Brown University, Providence, RI 02860, USA; hongwei_yao@brown.edu (H.Y.); abigail_peterson@brown.edu (A.L.P.); 2 Department of Epidemiology, Brown University, Providence, RI 02860, USA; jie_li@brown.edu (J.L.); Haiyan_Xu@brown.edu (H.X.); 3 Department of Pediatrics, Warren Alpert Medical School of Brown University, Providence, RI 02860, USA

**Keywords:** heme oxygenase, insulin sensitivity, glucose tolerance, energy expenditure, white adipose tissue, brown adipose tissue

## Abstract

Heme oxygenase (HO) consists of inducible (HO-1) and constitutive (HO-2) isoforms that are encoded by *Hmox1* and *Hmox2* genes, respectively. As an anti-inflammatory and antioxidant molecule, HO participates in the development of metabolic diseases. Whether *Hmox* deficiency causes metabolic abnormalities under basal conditions remains unclear. We hypothesized that HO-1 and HO-2 differentially affect global and adipose tissue metabolism. To test this hypothesis, we determined insulin sensitivity, glucose tolerance, energy expenditure, and respiratory exchange ratio in global *Hmox1^-/-^* and *Hmox2^-/-^* mice. Body weight was reduced in female but not male *Hmox1^-/-^* and *Hmox2^-/-^* mice. Reduced insulin sensitivity and physical activity were observed in *Hmox1^-/-^* but not *Hmox2^-/-^* mice. Deletion of either *Hmox1* or *Hmox2* had no effects on glucose tolerance, energy expenditure or respiratory exchange ratio. Mitochondrial respiration was unchanged in gonadal fat pads (white adipose tissue, WAT) of *Hmox1^-/-^* mice. *Hmox2* deletion increased proton leak and glycolysis in gonadal, but not interscapular fat tissues (brown adipose tissue, BAT). Uncoupling protein and *Hmox1* genes were unchanged in gonadal fat pads of *Hmox2^-/-^* mice. Conclusively, HO-1 maintains insulin sensitivity, while HO-2 represses glycolysis and proton leak in the WAT under basal condition. This suggests that HO-1 and HO-2 differentially modulate metabolism, which may impact the metabolic syndrome.

## 1. Introduction

Heme oxygenase (HO) consists of inducible (HO-1) and constitutive (HO-2) isoforms that are encoded by *Hmox1* and *Hmox2* genes, respectively. HO-1 is ubiquitously expressed in many organs, including the lung, heart, skeletal muscle, kidney, and spleen. It is also expressed in metabolic organs, including the liver and white adipose tissue [1]. HO-2 is highly expressed in the testis and brain [2,3]. As an Nrf2-dependent gene, HO-1 protein can modulate its own gene expression in a feed forward transcriptional manner [4]. In addition, HO-1 can interact with and modulate Nrf2 activation, setting an adaptive reprogramming that enhances antioxidant defenses [5]. Although HO-2 is constitutively expressed, the *Hmox2* gene can also be regulated by glucocorticoids through the glucocorticoid responsive element in rat testis [2]. HO catalyzes the degradation of heme yielding cytoprotective products, including bilirubin, ferritin, and carbon monoxide. Heme, an iron-protoporphyrin IX, is an essential co-factor involved in multiple biological processes, including oxygen transport and storage, electron transfer, drug and steroid metabolism, and signal transduction. However, excess free-heme is highly toxic due to its ability to promote oxidative stress. HO products have been shown to suppress oxidative stress and inflammatory responses. Therefore, HO-1 is considered as a classical component of stress response to a variety of chemical and physical stimuli, and HO functions as a cytoprotective molecule [6,7].

Accumulating evidence shows that HO by-products also participate in glucose metabolism as well as in the development of metabolic syndrome and disorders. This syndrome is characterized by obesity, hyperglycemia, dyslipidemia, and insulin resistance concomitant with other abnormalities, including hypertension. A sedentary lifestyle, physical inactivity, and poor eating habits are aggravating factors [8,9]. For instance, serum bilirubin levels are lower in overweight asymptomatic middle-aged adults [10]. Elevated ferritin for iron stores is positively associated with the prevalence of metabolic syndrome and with insulin resistance [11]. Carbon monoxide induces metabolic switch in adipocytes and reduces insulin resistance in obese mice [12]. Administration of hemin, an HO-1 inducer, protects against streptozotocin-induced increase in blood glucose in rats [13]. This suggests that HO may play an important role in modulating glucose and energy metabolism. In fact, HO activity is increased after exercise in healthy volunteers [14], but is reduced in obese mice compared with age-matched lean mice [15]. Pharmacological administration of the HO-1 inducer cobalt protoporphyrin reduces weight gain, visceral and subcutaneous fat content, and insulin levels but improves insulin sensitivity and glucose tolerance in obese mice [15]. This is associated with increased adiponectin and reduced inflammatory responses. In contrast, inhibition of HO activity by stannous mesoporphyrin decreases adiponectin and increases secretion of inflammatory cytokines in obese mice [15]. Nevertheless, whether HO deficiency causes metabolic abnormalities under basal conditions remains unclear.

We hypothesized that HO-1 and HO-2 differentially affect global and adipose tissue metabolism. To test this hypothesis, we first determined insulin sensitivity, glucose tolerance, energy expenditure, and respiratory exchange ratio in *Hmox1* knockout (*Hmox1^-/-^*) and *Hmox2^-/-^* mice as well as mitochondrial respiration and glycolysis in white adipose tissue (WAT). Metabolic dysregulation is associated with low physical activity and leads to cognitive decline [16,17]. We, therefore, also evaluated physical activity and motor coordination in these knockout mice compared to their wild-type (WT) littermates.

## 2. Results

### 2.1. Body Weight Was Reduced in Female Hmox1^-/-^ and Hmox2^-/-^ Mice Compared to Their Female WT Littermates

**Figure 1:**
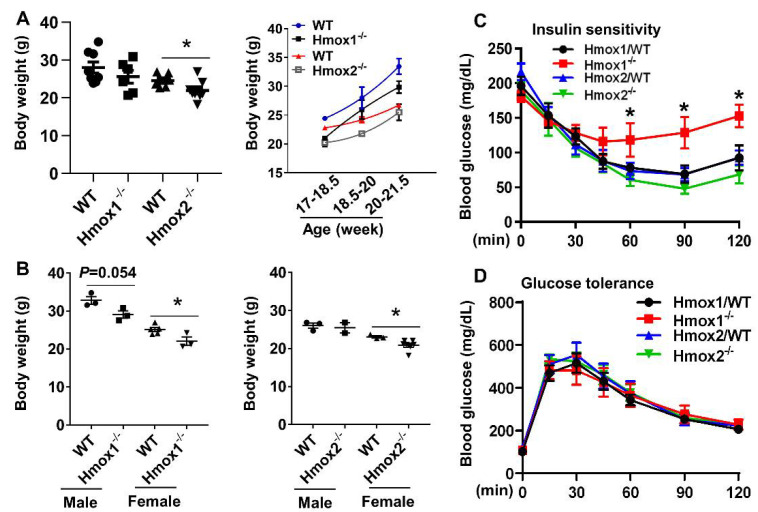
Insulin sensitivity was reduced in *Hmox1*^-/-^ but not in *Hmox2*^-/-^ mice. *Hmox1*^-/-^, *Hmox2*^-/-^ mice and their wild-type (WT) littermates (4–5 months old) were used for detecting insulin sensitivity and glucose tolerance. (**A**,**B**) Body weight of *Hmox1*^-/-^, *Hmox2*^-/-^ mice and their WT littermates were assessed. (**A**) Left panel denotes body weight of mixed male and female mice, while right panel denotes the changes in body weight with different ages. (**C**) Insulin (1.25 U/kg for females, 2 U/kg for males) was intraperitoneally injected, and blood glucose was measured at 0, 15, 30, 45, 60, 90, and 120 min post injection. (**D**) Glucose (2 g/kg) was intraperitoneally injected, and blood glucose was measured at 0, 15, 30, 45, 60, 90, and 120 min post injection. Data are expressed as mean ± SEM. *n* = 7–9. * *p* < 0.05 vs. corresponding WT littermates.

We assessed body weight of *Hmox1^-/-^* and *Hmox2^-/-^* mice as well as their WT littermates at 4–5 months of age to determine whether the mice had early evidence of obesity. In agreement with a previous study [18], body weight was not altered in *Hmox1^-/-^* mice when data were mixed from male and female mice. We then compared body weight in male and female knockout mice separately. There were no differences in body weight of male *Hmox1^-/-^* and *Hmox2^-/-^* mice compared to their male WT littermates, although sample sizes were small in certain groups (Figure 1A). However, body weight was reduced in both female *Hmox1^-/-^* and *Hmox2^-/-^* mice compared to their female WT littermates (Figure 1B). Although there were no differences in age among these groups (Yao H, Peterson AL, Dennery PA. Brown University, Providence, RI. Heme oxygenase/metabolism. 2020), *Hmox2*^-/-^ mice grew faster than their WT littermates at 4–5 months old (Figure 1A). These results demonstrate that deletion of *Hmox1* and *Hmox2* affects body weight in a gender-specific manner but does not suggest obesity.

### 2.2. Insulin Sensitivity Was Reduced in Hmox1^-/-^ but not in Hmox2^-/-^ Mice

We determined whether deletion of *Hmox1* and *Hmox2* alters insulin sensitivity by injecting insulin and measuring blood glucose levels within 2 h post injection. As shown in Figure 1C, as expected, in WT mice, blood glucose gradually decreased after insulin injection, whereas it remained elevated in *Hmox1^-/-^* mice, which denotes loss of insulin sensitivity. In contrast, there were no differences in insulin sensitivity between *Hmox2^-/-^* and WT littermates. We then intraperitoneally injected glucose and measured blood glucose levels for a 2 h period post injection to assess glucose tolerance. As shown in Figure 1D, there were no changes in glucose tolerance among *Hmox1^-/-^* and *Hmox2^-/-^* mice compared to their WT littermates. No sex differences in insulin sensitivity or glucose tolerance were observed in both *Hmox1^-/-^* and *Hmox2^-/-^* mice compared to their WT littermates (Yao H, Peterson AL, Dennery PA. Brown University, Providence, RI. Heme oxygenase/metabolism. 2020). These results demonstrate that *Hmox1* deletion decreases insulin sensitivity but does not affect glucose tolerance under basal conditions. As for *Hmox2*, its deletion has no effect on glucose tolerance or insulin sensitivity.

### 2.3. Deletion of Hmox1 But Not Hmox2 Reduced Times for Active Behaviors

**Figure 2:**
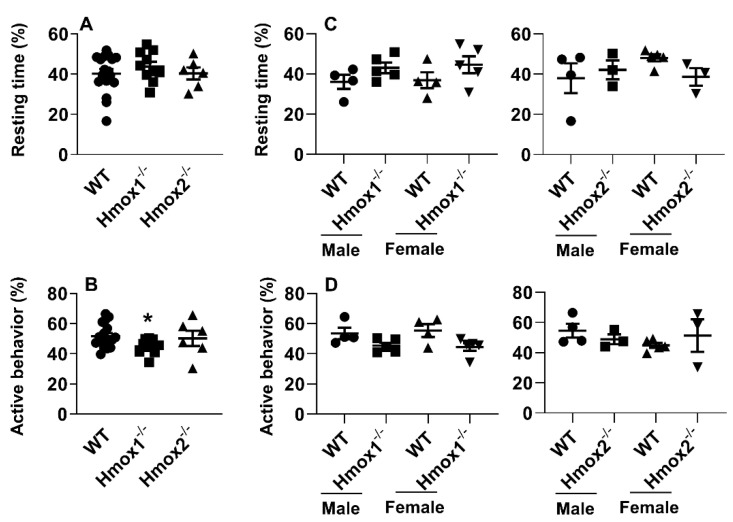
Physical activity was reduced in *Hmox1^-/-^* but not in *Hmox2^-/-^* mice. Physical activity was assessed in *Hmox1^-/-^*, *Hmox2^-/-^* mice and their WT littermates (4–5 months old) using continuous home cage video monitoring. (**A**,**C**) Percentage of resting time was calculated. (**B**,**D**) The sum of walking, hanging, grooming, and rearing was recorded as active behavior. Data are expressed as mean ± SEM. *n* = 6–10. * *p* < 0.05 vs. corresponding WT group.

To determine the effects of HO on physical activity, we utilized continuous home cage video monitoring to measure times spent on resting and active behaviors [19,20]. As shown in Figure 2A,B, compared to WT littermates, *Hmox1*^-/-^ mice had reduced times spent on active behaviors but not for resting. In the *Hmox2^-/-^,* there were no changes in times spent on resting or active behaviors compared to WT littermates (Figure 2A,B). No sex differences in physical activity were observed in both *Hmox1^-/-^* and *Hmox2^-/-^* mice compared to their WT littermates (Figure 2C,D). These results demonstrate that times of active behaviors are reduced in *Hmox1*^-/-^ but not *Hmox2*^-/-^ mice.

### 2.4. Motor Coordination Was Not Altered in Hmox1^-/-^ or Hmox2^-/-^ Mice

**Figure 3:**
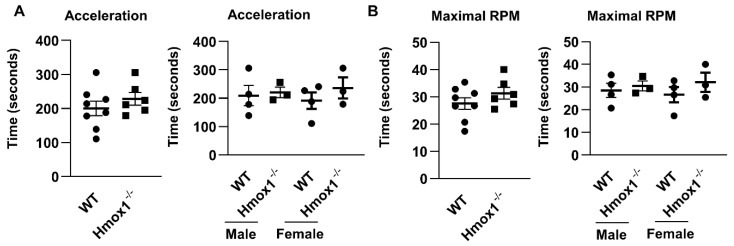
No changes in motor coordination between *Hmox1^-/-^* mice and WT littermates. Motor coordination was measured using the rotarod test in *Hmox1^-/-^* mice and WT littermates (4–5 months old). Riding time was recorded when the rotarod was hold accelerated (acceleration, **A**) or at the maximal speed (**B**). Data are expressed as mean ± SEM. *n* = 6–8.

The rotarod test is commonly used to assess motor coordination and balance in rodents [21]. High fat-feeding significantly accelerates decline in rotarod performance in mice [22]. As shown in Figure 3, there were no changes in the motor coordination or balance in terms of time before dropping from the rod or the maximal speed attained between *Hmox1^-/-^* mice and WT littermates. There were no sex differences in physical activity between *Hmox1^-/-^* mice and WT littermates (Figure 3). Similarly, there were no significant differences in motor coordination between *Hmox2^-/-^* and WT mice at 4–6 months old [23]. These results indicate that HO deficiency alone has no effects on motor coordination.

### 2.5. No Changes in Energy Metabolism or Respiratory Exchange Ratio in Either Hmox1 or Hmox2 knockout Mice

**Figure 4:**
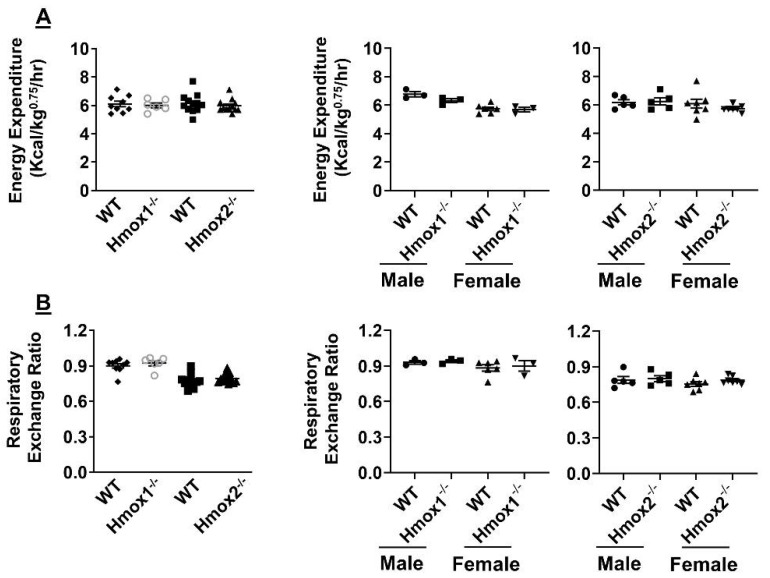
No changes in energy expenditure or respiratory exchange ratio in *Hmox1^-/-^* and *Hmox2^-/-^* mice. Energy expenditure and respiratory exchange ratio was measured using the metabolic chamber in *Hmox1^-/-^*, *Hmox2^-/-^* mice and their WT littermates (4–5 months old). Energy expenditure (**A**) and respiratory exchange ratio (**B**) were calculated. Data are expressed as mean ± SEM. *n* = 6–12.

Dysregulated energy metabolism is essential to the etiopathogenesis of metabolic syndrome [16,24,25]. Hence, we employed a metabolic chamber to evaluate energy expenditure and respiratory exchange ratio in both *Hmox1^-/-^* and *Hmox2^-/-^* mice. As shown in Figure 4, energy expenditure and respiratory exchange ratio were unchanged in either *Hmox1^-/-^* or *Hmox2^-/-^* mice compared to their WT littermates. There were no sex differences in energy expenditure or respiratory exchange ratio in both *Hmox1^-/-^* and *Hmox2^-/-^* mice compared to their WT littermates (Figure 4). These results suggest that HO does not affect energy metabolism in mice.

### 2.6. No Changes in Mitochondrial Respiration in WAT of Hmox1^-/-^ Mice, While Hmox2 Deletion Increased Proton Leak and Glycolysis in WAT

**Figure 5:**
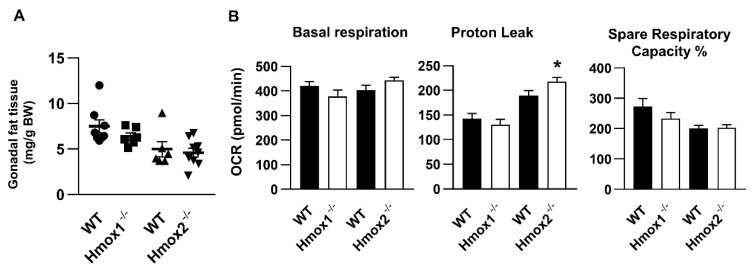
Mitochondrial respiration in gonadal white adipose tissue (WAT) of *Hmox1^-/-^* and *Hmox2^-/-^* mice. (**A**) Gonadal fat pads were weighted in *Hmox1^-/-^*, *Hmox2^-/-^* mice and their WT littermates (4–5 months old). (**B**) Mitochondrial oxidative phosphorylation was measured using a Seahorse Analyzer in gonadal fat pads from *Hmox1^-/-^*, *Hmox2^-/-^* mice and their WT littermates. Data are expressed as mean ± SEM. *n* = 6–10. * *p* < 0.05 vs. corresponding WT group.

The WAT plays an essential role in regulating systemic energy homeostasis [26]. The main WAT pads in the mouse are the inguinal and gonadal regions, with the latter being the most frequently utilized in experiments [27,28]. We, thus, normalized the weight of gonadal fat pads to body weight. As shown in Figure 5A, there were no differences in the ratio of WAT/body weight in both *Hmox1^-/-^* and *Hmox2^-/-^* mice compared to their corresponding WT littermates. We then determined WAT mitochondrial oxidative phosphorylation using an XF24 Seahorse Analyzer in the *Hmox1^-/-^* and *Hmox2^-/-^* mice. As shown in Figure 5B, mitochondrial respiration, including basal respiration, proton leak and spare respiratory capacity, was unchanged in gonadal fat pads of *Hmox1^-/-^* mice compared to WT littermates. Levels of proton leak were increased in gonadal fat pads of *Hmox2^-/-^* mice compared to WT littermates. There were no changes in basal respiration or spare respiratory capacity of WAT between *Hmox2^-/-^* mice and WT littermates (Figure 5B). The results demonstrate that *Hmox2* deletion increases proton leak to dissipate energy as heat or reduce ATP synthesis.

**Figure 6:**
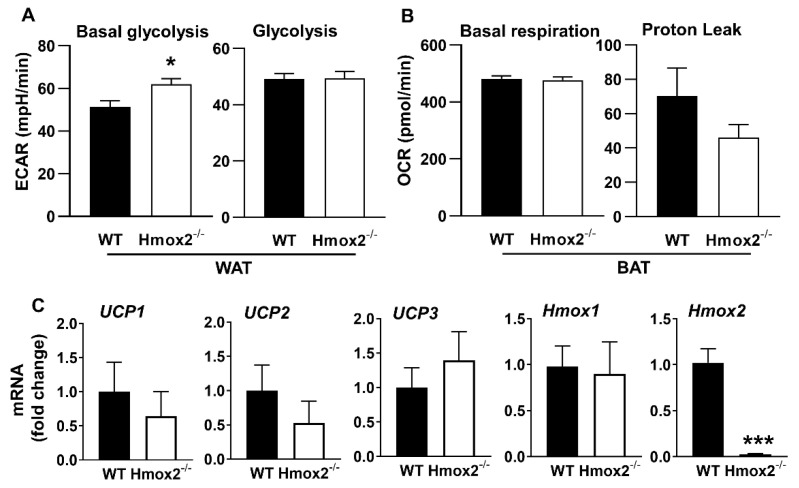
Glycolysis in gonadal WAT and mitochondrial respiration in interscapular brown adipose tissue (BAT) of *Hmox2^-/-^* mice. Glycolysis (**A**) and mitochondrial oxidative phosphorylation (**B**) were measured using a Seahorse Analyzer in gonadal and interscapular fat pads, respectively, from *Hmox2^-/-^* mice and WT littermates. (**C**) Uncoupling protein (UCP)*1*, *UCP2, UCP3, Hmox1*, and *Hmox2* gene expression were measured by qRT-PCR in gonadal WAT of WT and *Hmox2^-/-^* mice. Data are expressed as mean ± SEM. *n* = 5–7. * *p* < 0.05, *** *p* < 0.001 vs. WT group.

Since proton leak was increased in WAT of *Hmox2^-/-^* mice, we also measured glycolysis in gonadal fat pads of these mice. Basal glycolysis and glycolysis were calculated as extracellular acidification (ECAR) before (basal) and after (glycolysis) glucose injection during the glycolysis stress test. As shown Figure 6A, basal glycolysis was increased in WAT from *Hmox2^-/-^* mice compared to WT littermates. Brown adipose tissue (BAT) contains many more mitochondria than does the WAT. Thus, we also measured whether there were any changes in mitochondrial respiration in the interscapular fat pads of *Hmox2^-/-^* mice, which contain BAT. As shown in Figure 6B, basal respiration and proton leak were not changed in the BAT of *Hmox2^-/-^* mice compared to WT littermates. Altogether, *Hmox1* deletion has no effect on mitochondrial respiration, while *Hmox2* deletion increased proton leak and glycolysis in the WAT.

### 2.7. No Changes in Uncoupling Protein (UCP) or Homx1 Genes in Gonadal Fat Pads of Hmox2^-/-^ Mice

To determine whether increased proton leak in gonadal fat pads of *Hmox2*^-/-^ is associated with changes in UCP, we measured *UCP1*, *UCP2* and *UCP3* gene expression. As shown in Figure 6C, there were no changes in *UCP1*, *UCP2* or *UCP3* gene expression in gonadal fat pads of *Hmox2*^-/-^ mice compared to WT littermates. In addition, *Homx1* gene expression was unchanged in gonadal fat pads of *Hmox2*^-/-^ mice compared to WT littermates (Figure 6C). These results suggest that there were no compensatory changes in *Hmox1* gene in *Hmox2*^-/-^ mice, and *Hmox2* deletion-mediated metabolic changes in gonadal fat pads are not associated with *UCP* gene expression.

## 3. Discussion

HO has been implicated in the development of metabolic diseases, including obesity, diabetes, and hepatic steatosis [1,29,30]. Here we showed that body weight was reduced in both female *Hmox1^-/-^* and *Hmox2^-/-^* mice. In addition, *Hmox1^-/-^* mice spontaneously developed insulin resistance and had reduced physical activity. This was not observed in *Hmox2* knockout mice. Neither genotype had changes in energy expenditure or respiratory exchange compared to their WT littermates. Since both isoforms of HO catabolize the same enzymatic reaction, it is unlikely that the effect of *Hmox1* deletion on insulin resistance is tied to heme metabolism.

HO-1 expression in visceral adipose tissue (WAT) negatively correlates with waist-to-hip ratio and insulin resistance in humans [31]. This is supported by a study showing that HO-1 levels are reduced in adipocytes isolated from visceral fat tissues of obese mice compared to lean mice [30]. Ablation of *Hmox1* in adipose tissue increases pro-inflammatory visceral fat and abrogates a beige-cell like phenotype, a comparable thermogenic potential to brown fat cells [32]. This is in contrast to our findings that global deletion of *Hmox1* had no effect on the weight of gonadal fat tissue (WAT). This suggests a fat type-specific role of HO-1. Additionally, overexpression of *Hmox1* in adipose tissue does not protect against high fat diet-induced insulin resistance in mice [33], whereas in streptozotocin-induced diabetic rats, chronic induction of HO-1 by hemin reduces hyperglycemia and improves glucose metabolism [13]. This may suggest that HO-1 is not instrumental in diet-induced insulin resistance but may play a larger role in a more severe model of diabetes. Interestingly, in an age- and body mass index-matched cohort, *Hmox1* expression and HO-1 protein were augmented in livers and visceral fat of insulin-resistant, compared to insulin-sensitive, obese patients [1]. This suggests that HO-1 drives meta-inflammation and insulin resistance. We noticed that the insulin activity was intact based on the normal glucose tolerance test and the early response upon insulin challenge (first 30 min) in *Hmox1^-/-^* mice, compared to WT mice. In mice, conditional knockout of *Hmox1* in hepatocytes or macrophages does not affect liver morphology, basal blood glucose, or glucose tolerance [1]. This is in agreement with our findings that global knockout of *Hmox1* does not influence glucose tolerance. Insulin sensitivity can be improved by exercise and the latter has been shown to increase HO-1 activity [14,34,35]. Thus, reduced physical activity could have contributed to the reduced insulin sensitivity in *Hmox1^-/-^* mice in our experiments. The reduced late insulin response in *Hmox1^-/-^* mice could be induced by insulin antagonistic pathways in response to hypoglycemia such as hepatic gluconeogenesis, alterations in the hypothalamic–pituitary–adrenal axis and increased inflammatory cytokines [36,37]. We did not explore these possibilities here. Overall, the mechanisms underlying impaired insulin signals in *Hmox1^-/-^* mice remain unclear, especially when paired with normal glucose tolerance.

HO-1 is considered as a potential biomarker in Alzheimer’s disease and mild cognitive impairment [38]. Overexpression of *Hmox1* causes cognitive decline by regulating tauopathy in mice [39]. In aging rats, pharmacological induction of HO-1 attenuates cognitive decline and neuropathological alterations [34]. Further studies would be needed to fully understand spatial and motor performance in aging *Hmox1^-/-^* and *Hmox2^-/-^* mice.

*Hmox2* null mice on a C57BL/6 ×129/Sv genetic background display an increase in body weight as well as visceral and subcutaneous fat content compared to WT animals [40,41,42]. In contrast, we saw reduced body weight particularly in female mice and no changes in the ratio of gonadal fat to body weight in *Hmox2^-/-^* mice on a C57BL/6 background. This suggests that the genetic background modifies body weight and adipose phenotype in *Hmox2^-/-^* mice or that the small size of the *Hmox2^-/-^* initially may lead to later obesity and metabolic dysregulation [43,44]. In fact, others have shown that *Hmox2^-/-^* mice develop the metabolic syndrome at 8 months of age, including obesity, insulin resistance, and hypertension [41]. However, at 4–5 months of age, we saw no changes in insulin sensitivity, glucose intolerance or energy expenditure in *Hmox2*^-/-^ mice compared to WT littermates. This suggests that *Hmox2*^-/-^ mice develop the metabolic syndrome in an age-dependent manner, consistent with the onset of the metabolic syndrome in individuals who are small for gestational age or intrauterine growth restricted [45]. Although we did not document the weight of the pups at birth in each litter, *Hmox2*^-/-^ mice grew faster than their WT littermates at 4–5 months old. This may be responsible for the metabolic derangement in these knockout mice. We also did not examine blood pressure or lipid metabolism in the *Hmox1^-/-^* and *Hmox2^-/-^* mice at 4–5 months of age.

The UCP protein is capable of dissipating the proton gradient into mitochondrial matrix, called proton leak. The WAT does not normally exhibit uncoupled respiration or express UCP1. This agrees with high cycle threshold values observed in real-time PCR assay of *UCP1* in gonadal fat pads. Elevated UCP1 protein levels are sufficient to improve glucose uptake in human white adipocytes, whereas UCP2 protein represses glycolysis in fibroblasts [46,47]. In addition, lactate can increase *UCP1* and thermogenic gene expression as well as the browning of white adipocytes [48]. Nevertheless, increased proton leak and glycolysis in in gonadal fat pads of *Hmox2*^-/-^ mice are not associated with *UCP* expression. Whether increased proton leakage of WAT compensates for the abolishment of HO-2 activity in nearby tissues is unclear. HO-2 coordinates with 6-phosphofructo-2-kinase/fructose-2,6-bisphosphatase 4, a key enzyme in glycolysis, to maintain glucose homeostasis [49], however its role in our model was not explored. Ultimately, it is still unknown by which pathways *Hmox2* deletion increases glycolysis. Overall, the increased proton leak and increased glycolysis would contribute to less generation of ATP and increased heat loss, possibly explaining the lower weights in the *Hmox2*^-/-^ females.

Usually there is compensatory induction of HO-1 in the absence of HO-2, but not vice versa [50,51]. Interestingly, HO-1 protein is decreased in *Hmox2^-/-^* adipocytes, and pharmacological induction of HO-1 by cobalt protoporphyrin rescues adipocyte dysfunction in *Hmox2^-/-^* mice [52]. We did not observe changes in *Hmox1* gene expression in *Hmox2^-/-^* mice. Thus, abnormal proton leak and glycolysis in *Hmox2^-/-^* mice seems not due to changes in HO-1. In contrast to the changes in the gonadal WAT, there were no changes in proton leak in the interscapular BAT of *Hmox2^-/-^* mice. The discrepancies in proton leak between WAT and BAT in *Hmox2^-/-^* mice remain unclear.

There are certain limitations in this study, which include an unbalanced and small sample size in certain groups. This particularly affects the analysis of statistical power for the gender subgroups.

In conclusion, reduced insulin sensitivity and physical activity were observed in *Hmox1^-/-^* but not in *Hmox2^-/-^* mice. There were no changes in energy expenditure, spatial and motor performance deficits, gonadal fat weight, or mitochondrial respiration of gonadal fat tissues in young adult *Hmox1^-/-^* mice. Gonadal fat weight, physical activity, and energy expenditure were not altered by *Hmox2* deletion. Both proton leak and glycolysis were increased in gonadal fat tissues in young adult *Hmox2^-/-^* mice, which was not associated with changes in *UCP* expression.

## 4. Materials and Methods

### 4.1. Mice

*Hmox1^-/-^* and *Hmox2^-/-^* mice as well as their WT littermates (male and female, 4–5 months old) were used as we described previously [53,54]. *Hmox1^-/-^* mice are of a C57Bl/6 background. We crossed *Hmox2^-/-^* mice (B6/129 background) with C57BL/6 mice to generate *Hmox2^-/-^* mice with a C57BL/6 background for experiments. All animal experiments were reviewed and approved by the Institutional Animal Care and Use Committee (IACUC) of Brown University. The number of the IACUC is 1507000150, which was approved on 1 October 2015.

### 4.2. Glucose Tolerance and Insulin Sensitivity Testing

For glucose tolerance tests, mice were fasted for 16 h. A baseline glucose reading was taken using a handheld glucose meter, followed by an intraperitoneal injection of glucose (2 g/kg) [55]. Glucose readings were taken at 15, 30, 45, 60, 90, and 120 min post-injection. For insulin sensitivity tests, mice were fasted for 6 h. A baseline glucose reading was taken using a handheld glucose meter, followed by an injection of insulin (1.25 U/kg for females, 2 U/kg for males, Humulin-R, Lilly) [56]. Different doses of insulin were used in male and female, as female mice are more insulin sensitive than males [57]. Glucose readings were taken at 15, 30, 45, 60, 90, and 120 min post insulin injection.

### 4.3. Home Cage Video Monitoring

Mice were placed in individual shoebox-style cages and allowed to acclimate to the cages for 48 h before monitoring began. Mice were kept on a normal light/dark cycle and provided food and water ad libitum. Mouse spontaneous activity in home cage was recorded using automated computer vision analysis (CLAMS, Oxymax Open Circuit Calorimeter, Columbus Instruments, Columbus, OH, USA) of continuous video recordings for 5 days [58]. The activity includes micromovements, sleeping/resting, consumption (eating and drinking), and active behaviors (grooming, hanging, rearing and walking). The percentage of time spent on resting and active behaviors was presented.

### 4.4. Rotarod Testing

Motor coordination in mice was tested using a rotarod instrument (MedAssociates, Inc., St. Alban, VT) as previously described [59]. In brief, mice were acclimated to the rotarod at 4 revolutions per min (RPM) for 1 min prior to experiments. For testing, the instrument accelerated at a constant rate from 4 to 40 RPM over 5 min. The latency to fall off during acceleration and at maximal speed (40 RPM) was recorded digitally when the mouse fell from the rotarod and broke an infrared beam. Mice were tested three times per day on three consecutive days, and the best performance for each animal was taken.

### 4.5. Measuring Indirect Calorimetry

A Comprehensive Laboratory Animal Monitoring System (Columbus Instruments) was used to measure mouse oxygen consumption (VO_2_), CO_2_ production (VCO_2_), and respiratory quotient as described previously [56,60]. Mice were placed in individual boxes with food and water provided ad libitum. Mice were allowed to acclimate to the chambers for 24 h before data collection began, and data was collected over a 24 h period. Energy expenditure was calculated using the formula VO_2_ × (3.815 + 1.232 × respiratory quotient), and normalized to (body mass)^0.75^ [56]. Respiratory exchange ratio was calculated as a ratio of VCO_2_ toVO_2_.

### 4.6. Evaluating Mitochondrial Respiration and Glycolysis in Whole Adipose Tissues

Mitochondrial respiration and glycolysis were measured in adipose tissues using a Seahorse XF24 Analyzer (Agilent Technologies) as described previously [61]. In brief, we used a 3 mm biopsy punch to harvest a plug of tissue from gonadal (white) and interscapular (brown) fat pads. Adipose tissue (~20 mg) was placed to cover the center of the wells of the islet capture plate. Islet capture screens were applied and wells were washed twice with warm assay medium to remove fat droplets. Tissue was equilibrated for 8 baseline measurements. After the first and second injections, 5 measurements were taken, and after the final injection, 7 measurements were taken. Drug concentrations in ports were as follows: oligomycin: 100 μM, FCCP: 800 μM, rotenone: 30 μM, antimycin A: 120 μM, glucose: 200 mM, and 2-DG: 1.25 M.

### 4.7. Determining mRNA Levels by qRT-PCR

Total RNA was extracted by the TRIzol reagent, and purified using the RNeasy miniprep kit (Qiagen, Valencia, CA, USA). RNA samples were quantified by the NanoDrop™ One Microvolume UV-Vis Spectrophotometer (Thermo Scientific, Wilmington, DE, USA). Then, 400 nanograms of total RNA was used for reverse transcription with Taqman^®^ Reverse Transcription Reagents (ThermoFisher Scientific). One microliter of cDNA was used for real-time PCR reactions in the 7300 Real-Time PCR System (Applied Biosystems). All Taqman gene probes were purchased from Thermo Fisher Scientific: *UCP1* (Mm01244861_m1), *UCP2* (Mm00627599_m1), *UCP3* (Mm01163394_m1), *Hmox1* (Mm00516006_m1) and *Hmox2* (Mm00468922_m1). Gene expression was normalized to 18s rRNA levels. Relative RNA abundance was quantified by the comparative 2^−ΔΔ*C*t^ method.

### 4.8. Statistical Analysis

Statistical analyses were performed using GraphPad Prism 8 (GraphPad Software, San Diego, CA, USA). The results were expressed as mean ± SEM. The *t*-test was used for detecting statistical significance of the differences between means of two groups after checking the normality of data. The statistical significance of the differences among groups was evaluated by using two-way ANOVA for overall significance, followed by the Tukey–Kramer test. Statistical significance was considered present when *p* < 0.05.

## 5. Conclusions

HO-1 maintains insulin sensitivity, while HO-2 represses glucose metabolism and proton leak in white adipose tissues under basal condition. This suggests that HO-1 and HO-2 differentially modulate global metabolism as well as metabolism of adipose tissues.

## Abbreviation

BAT	brown adipose tissue;
ECAR	extracellular acidification;
HO	heme oxygenase;
OCR	oxygen consumption rate;
UCP	uncoupling protein;
WAT	white adipose tissue;
